# Trait-trait dynamic interaction: 2D-trait eQTL mapping for genetic variation study

**DOI:** 10.1186/1471-2164-9-242

**Published:** 2008-05-23

**Authors:** Wei Sun, Shinsheng Yuan, Ker-Chau Li

**Affiliations:** 1 Department of Biostatistics, University of North Carolina, Chapel Hill, NC, 27599, USA; 2 Department of Genetics, University of North Carolina, Chapel Hill, NC, 27599, USA; 3 Carolina Center for Genome Science, University of North Carolina, Chapel Hill, NC, 27599, USA; 4 Institute of Statistical Science, Academia Sinica, Taipei, 115, Taiwan, China; 5 Department of Statistics, University of California, Los Angeles, CA, 90095, USA

## Abstract

**Background:**

Many studies have shown that the abundance level of gene expression is heritable. Analogous to the traditional genetic study, most researchers treat the expression of one gene as a quantitative trait and map it to expression quantitative trait loci (eQTL). This is 1D-trait mapping. 1D-trait mapping ignores the trait-trait interaction completely, which is a major shortcoming.

**Results:**

To overcome this limitation, we study the expression of a pair of genes and treat the variation in their co-expression pattern as a two dimensional quantitative trait. We develop a method to find gene pairs, whose co-expression patterns, including both signs and strengths, are mediated by genetic variations and map these 2D-traits to the corresponding genetic loci. We report several applications by combining 1D-trait mapping with 2D-trait mapping, including the contribution of genetic variations to the perturbations in the regulatory mechanisms of yeast metabolic pathways.

**Conclusion:**

Our approach of 2D-trait mapping provides a novel and effective way to connect the genetic variation with higher order biological modules via gene expression profiles.

## Background

**Figure 1:**
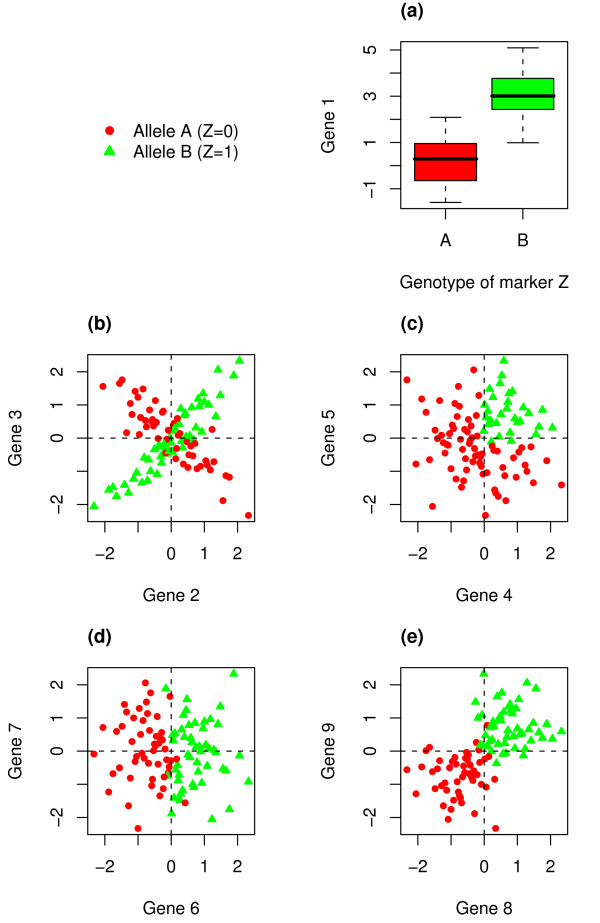
**A schematic diagram of eQTL studies**. Suppose the eQTL is captured by marker *Z *with allele A and B. We code Z as 0 or 1 if the inherited allele is A or B, respectively. (a) 1D-trait mapping: conventional eQTL mapping compares one gene expression profile against one marker genotype profile to find significant differential expression. (b) 2D-trait mapping: co-expression trait mapping aims at the detection of changes in the co-regulation pattern by comparing two genes' expression profiles against one marker genotype profile. (c) Two genes are co-up-regulated under allele B(Z = 1). (d) The expression of one gene has a shift in the marginal distribution, detectable by 1D mapping. (e) The expression of both genes are shifted in the marginal distributions, detectable by 1D mapping. Only (b) and (c) are detectable by our 2D-trait mapping method.

Known as "eQTL" or "genetical genomics", the approach of treating gene expression profiles as quantitative traits and mapping them to genetic loci have been applied in yeast [1,2], worm [3], plant [4,5], fly [6], mouse [7,8], rat [9], and human [10,11] recently. These studies have shown that the level of gene expression is highly heritable, and it can be linked to either a local locus (cis-linkage) or a distant locus (trans-linkage). Most eQTL studies consider the expression profiles of different genes as different traits to be mapped one by one separately. The expression profile of a single gene can be compared with the genotype profiles of densely distributed genetic markers to find significant linkages/associations (Figure 1(a)). This is 1D (one dimension) trait mapping.

In this work, we shall investigate a different type of genetic interference that cannot be analyzed under the above one-gene one-trait formulation. Recent studies have demonstrated that transcription regulation of functionally associated genes can be dependent on the relevant cellular states such as fluctuations in the levels of nutrients, metabolites, hormones or other signaling molecules [12]. This raises the question of whether the co-regulation pattern, hence the co-expression pattern of two genes is affected by genetic variations. Figure 1(b) suggests a scenario where a DNA polymorphism captured by marker *Z *affects the co-expression pattern of a pair of genes Gene2 and Gene3. Suppose Z has two alleles, A and B. For the cells with allele *B*, Gene2 and Gene3 are seen to be positively co-expressed. But if the genetic variation (from *B *to *A*) affects the regulatory mechanism, we may observe weaker or even sign-changing correlation between Gene2 and Gene3. We shall develop a method to map the change of co-expression/correlation pattern of two traits to genetic loci, and refer it as 2D-trait mapping.

Our 2D-trait mapping approach is different from the traditional multivariate analysis of quantitative traits [13-15]. In these statistical multi-trait models, although different traits are correlated, the covariance between traits is assumed to be unaffected by genotypes. In other words, genotypes can only affect the multi-trait mean, but not the covariance. Thus multivariate trait analysis in the literature did not address the issue to be studied here.

The rest of the paper is organized as follows. In method section, we present our 2D-trait mapping approach and the eQTL data. In result section, we first carry out the full genome study on 16.5 millions of gene pairs, and then concentrate on gene pairs from metabolic pathways. We also use 2D-trait mapping to study how a transcription factor (TF) regulates the expression of its target genes. In discussion section, we summarize the merits of our approach and discuss some directions of generalization.

## Methods

### Data

We apply our 2D-trait mapping method to a yeast eQTL dataset [1], which includes data from a genetically variable population of 40 yeast segregants generated from a cross of two budding yeast strains: a standard laboratory strain (BY) and a wild isolate from a California vineyard (RM). Data for 6229 gene expression traits and 3313 single nucleotide polymorphism (SNP) markers are collected for each yeast segregant. The genotype profile of each marker is a binary vector, indicating from which parental strain the allele is inherited. The gene expression data is downloaded from Gene Expression Omnibus (GEO) [16]: GDS 91 and GDS 92 in series GSE37. The genotype data are downloaded from Leonid Kruglyak's laboratory's website [17]. The genotype profiles of neighboring markers tend to have very high correlations and some are even identical. We merge adjacent markers into marker blocks sequentially using the criterion that any two marker profiles within one block are either the same or different by only one segregant. The 3313 markers are merged into 667 marker blocks. The dichotomized centroid of all the markers within a marker block is used to represent the marker block [See section 1 of Additional file 1 for details].

An anonymous referee pointed out that, as an alternative to the marker block approach, traditional interval mapping approach where one estimates line origin at fixed intervals can be used to obtain continuous Z.

### Liquid Association for Binary Marker Profiles

Quantifying the co-expression pattern between two genes (2D trait) is more complicated than quantifying the expression level of one gene (1D trait). We employ the statistical concept "Liquid Association" (LA) [18] to address this 2D problem. Suppose *X*, *Y*, *Z *are continuous random variables with mean 0 and variance 1. Then the correlation between *X *and *Y *is just *E*(*XY*). LA aims to describe the change of the conditional expectation *g*(*z*) = *E*(*XY*|*Z *= *z*). If *Z *is a continuous random variable, change of the conditional expectation can be described by its derivative. This leads to the mathematical definition of LA:

(1) *LA*(*X*, *Y*|*Z*) = *E*(*g'*(*Z*))

A simple estimation of LA score is available if *Z *follows the standard normal distribution:

LA(*X*, *Y*|*Z*) = *E*(*XYZ*) [18]. To ensure normality, the normal quantile transformation is applied to each gene. This data pre-processing step is carried out after downloading the expression data from GEO. In 2D-trait mapping to binary markers, random variables *X *and *Y *represent the normalized gene expression profiles of two genes, while *Z *represents a marker genotype profile which only takes two possible values 0 and 1 and indicates from which parental strain the segregant inherits Z. We define Liquid Association score specifically for binary variable *Z *using a proper rescaling of *Z*. Assume *P*(*Z *= 1) = *a *and *P*(*Z *= 0) = *b *(*a *+ *b *= 1). We transform *Z *to *Z' *such that $Z^{\prime }=-\sqrt{a/b}$ if *Z *= 0; $Z^{\prime }=\sqrt{b/a}$ if *Z *= 1.

Under this transformation, *E*(*Z'*) = 0, *Var*(*Z'*) = 1, and LA(*X*, *Y*|*Z*) is given by

(2) $$E(XYZ^{\prime })=\sqrt{ab}[E(XY|Z=1)-E(XY|Z=0)]$$

Comparing equation (2) with equation (1), we see that "difference" is used to replace "derivative". The term $\sqrt{ab}$ can be viewed as a penalty for unbalance in the frequencies of two alleles of *Z*. It takes the maximum value if *a *= *b *= 1/2 (the case of Hardy-Weinberg equilibrium) and it gets smaller as the difference between *a *and *b *becomes larger. To compute (2), we replace *E*(*XY*|*Z *= 1) and *E*(*XY*|*Z *= 0) by the average of *XY *for all yeast segregants with Z = 1 and Z = 0 respectively.

LA can reflect the intuitive change in the co-expression/co-regulation of a pair of genes X and Y. Following most gene expression studies [19], the baseline expression of a gene is given by the average expression of all yeast segregants from the cross of RM strain and BY strain, which has been set to zero for each gene because of the normalization at the data preprocessing stage. A segregant with *X *> 0 and *Y *> 0 (*X *< 0 and *Y *< 0, respectively) is a case of an up-regulation (down-regulation, respectively) in both genes. Co-up-regulation or co-down-regulation contributes a positive value in the product XY. Likewise, contra-expression/contra-regulation (either "*X *> 0 and *Y *< 0", or "*X *< 0 and *Y *> 0") contributes a negative value in the product *XY*. Thus by calculating the average contribution to the product *XY *from segregants inheriting *Z *from one strain and compare it with that from segregants inheriting *Z *from another strain, we can quantify the change of co-regulation pattern. For the ideal scenario as depicted in Figure 1(b), *E*(*XY*|*Z *= 1) is positive because for *Z *= 1 (green dots) most cases are co-regulated; *E*(*XY*|*Z *= 0) is negative because for *Z *= 0 (red dots) most cases are contra-regulated; thus the net LA score is positive. LA can also detect patterns like Figure 1(c). But LA will not detect patterns like Figures 1(d), 1(e) where one or both genes are connected to *Z *only through 1D linkage. Specifically, for Figure 1(d), the expression of Gene 6 has a shift between Z = 0 and Z = 1. The contribution to *E*(*XY*|*Z *= 1) comes from both co-up-regulation and contra-regulation, which by and large cancels out each other because of symmetry. Similarly, the contribution to *E*(*XY*|*Z *= 0) comes from both co-down-regulation and contra-regulation, which again cancel out each other. Therefore LA score will be close to 0. In Figure 1(e), *E*(*XY*|*Z *= 1) and *E*(*XY*|*Z *= 0) cancel out each other because both are positive and take about the same size. Therefore LA score is close to 0 as well. In general, the size of LA score quantifies the degree of change in the co-expression pattern of a pair of genes as the genotype of a marker changes. The sign of the LA score indicates the direction of the change. Large LA scores, either positive or negative are of special interest.

### LA linkage score and P-value

For each fixed gene pair (*X*, *Y*), we find the marker blocks with the most positive and the most negative LA scores respectively and define two LA linkage scores, LA(*X*, *Y*)_max _= max_*Z*_*LA*(*X*, *Y*|*Z*) and LA(*X*, *Y*)_min _= min_*Z*_*LA*(*X*, *Y*|*Z*). We consider positive and negative LA linkage scores separately because they have different implications. Since alleles from strain BY and RM are coded as 1 and 0 respectively, a positive LA linkage score indicates that the gene pair has a higher correlation when the allele is inherited from strain BY than from strain RM; a negative LA linkage score indicates just the opposite way of correlation change. To determine if a LA linkage score is statistically significant or not, we address the issue of multiple testing across markers by a permutation p-value procedure. Specifically, we randomly permute the yeast segregant labels to generate a reference distribution for the LA linkage scores. At each permutation, we re-compute the LA scores for all markers and record the most positive and most negative values. After performing *M *permutations, *M *being a large number, we obtain the reference distribution of LA linkage scores that represents the no-linkage situation. The p-value for an observed LA linkage score is computed by dividing the number of LA linkage scores in the reference distribution that are higher (for positive score) or lower (for the negative score) than the observed LA linkage score by *M*. Now suppose there are altogether *N *gene pairs under consideration and the permutation p-value cutoff is *p*. We calculate the false discovery rate [20,21]: FDR = *Np*/*D*, where *D *is the number of gene pairs with permutation p-value ≤ p. Note that this is a conservative estimate of FDR by assuming the proportion of the null cases is 100%. The true proportion of null cases should be somewhat smaller but is in general not easy to estimate accurately despite of various suggestions in the literature. For more discussion on the complex issues concerning the estimation of null proportion in linkage studies, see [22].

## Results

### Genome-wide results of 2D-trait mapping

To gain a full genome view of 2D-trait mapping, we computed the positive LA linkage score and the negative LA linkage score for every gene pair and used them to rank the gene pairs. We examined gene pairs with exceptionally large LA scores, both positive and negative, and found many of them are functionally associated. For instance, from the first 20 gene pairs with the highest positive LA linkage scores [See Table S1 in Additional file 2], we found nine pairs linked to marker block 348. We observed that all but one gene are associated with functions of mitochondria. Among these genes, *CYC1 *appears 4 times, pairing with mitochondrial small (*MRPS28*, *RSM28*) and large (*MRPL22*) subunits and *YLR168C*, which encodes a putative protein of unknown function that may be involved in intra-mitochondrial sorting. *CYC1 *encodes cytochrome c, which serves as the electron carrier of the mitochondrial inter-membrane space that transfers electrons from ubiquinone-cytochrome c oxidoreductase to cytochrome c oxidase during cellular respiration.

**Figure 2:**
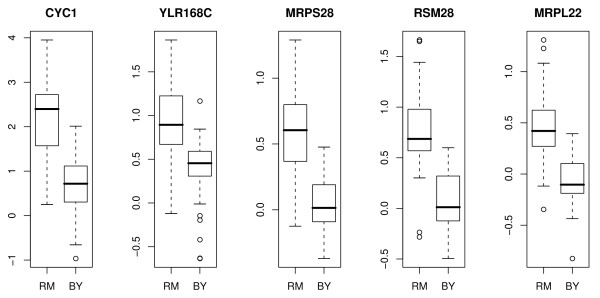
**1D-trait mapping result related with *CYC1***. 1D-trait mapping result for *CYC1 *and four LA-paired genes: *MRPS28*, *RSM28*, *MRPL22*, and *YLR168C*.

**Figure 3:**
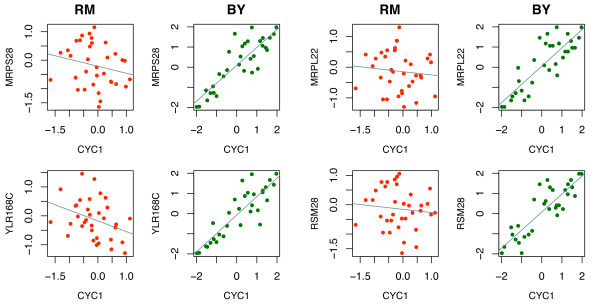
**1D-trait mapping result related with *CYC1***. 2D-trait mapping result for *CYC1 *and four LA-paired genes: *MRPS28*, *RSM28*, *MRPL22*, and *YLR168C*. Each green (red) dot indicates a yeast segregant of which the allele of marker block 348 is inherited from BY (RM) strain.

To elaborate the difference between 1D-trait mapping and 2D-trait mapping, we first examined the 1D-trait mapping result of these genes (Figure 2). *CYC1 *is trans-linked to Marker block 449 (ChrXII: 642137 to 663370), which contains the transcription factor Hap1 (ChrXII: 646417 to 650925) known to regulate the transcription of *CYC1*. On the other hand, by 1D-trait mapping, *MRPS28*, *RSM28*, *MRPL22*, and *YLR168C *are all trans-linked to marker block 550 at ChrXIV: 486861. We found the gene *TOM7 *(component of the translocase of outer membrane complex, TOM, responsible for recognition and initial import steps for all mitochondrially directed proteins) located nearby (ChrXIV: 493367 to 493549). The genotype profiles of marker blocks 449 and 550 have very low correlation (.06) because they are located in different chromosomes. Therefore while these results from 1D-trait mapping explain well the shifting of average gene expression due to the marker inherence from one yeast strain to another (Figure 2), they cannot explain co-regulation patterns between *CYC1 *and its LA paired genes. To see what additional gene regulation information that 2D-trait mapping can provide, we examined the makerblock 348 (ChrX: 336317 to 345059) and found a gene, *TIM54 *(component of the mitochondrial Tim54p-Tim22p complex involved in the insertion of polytopic proteins into the inner membrane), located at ChrX: 334260 to 335696. As shown in Figure 3, the correlation between *CYC1 *and its LA paired genes is very strong when the allele for the marker block 348 is inherited from the BY strain. In contrast, the positive correlation is lost when the allele is inherited from the RM strain.

We further studied the LA 2D-trait mapping results in a genome-wide scale. We obtained two genome-wide distributions of LA linkage scores, one for positive scores and one for negative scores, based on a total of 16.5 million gene pairs. Because an exceedingly large number of gene pairs are involved in the mapping and the majority of them are probably biologically unrelated, the LA linkage scores are highly susceptible to random chance. To help assess the degree of impact by chance fluctuation, we generated a reference distribution of linkage scores under the assumption of no linkage using a simulation scheme [See section A of Additional file 2]. Briefly, we first put all gene expression values into a data pool, ignoring the gene names and the segregant identities. We then simulated each gene profile by randomly sampling from the data pool with replacement. For a pair of random gene expression profiles, we computed two most extreme LA linkage scores (across all the 667 marker blocks), one for the positive value and another for the negative value. After repeating this procedure 1 million times, we obtained one reference distribution for the positive linkage scores and one for the negative LA linkage scores. We then compared the genome-wide LA score distribution with the reference distribution by quantile to quantile (Q-Q) plot. As expected, we found a global linear pattern [Figure S1 in Additional file 2]. The small but important difference is revealed by subtraction [Figure S2 in Additional file 2], where one can find an upward shifting of the quantiles for the genome-wide positive LA linkage scores and a downward-shifting for the negative scores. If the 0.001 quantile from the reference distribution is used to draw the cutoff point (i.e., p-value = 1e-3), we would detect 47,445 significant positive LA gene pairs and 49,646 significant negative LA gene pairs. The corresponding false discovery rates are 34.1% and 33.1% for positive and negative LA scores, respectively. When lowering the p-value cutoff to be 1e-5, we can reduce the FDR to 27.3% and 13.6% for positive and negative LA results respectively, which are still modest. But a clear message is that although the information content in the tail part of the LA linkage distribution is only modestly rich, useful results can still be detected. The results need to be subject to closer biological discern however [More discussion in section A of Additional file 2].

**Figure 4:**
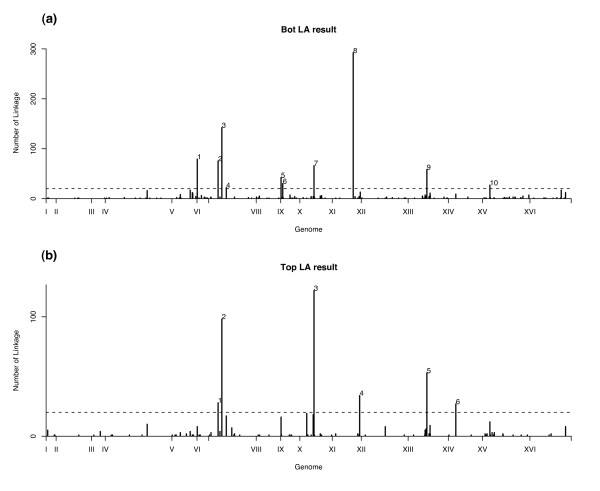
**Marker block frequency for bottom/top LA result**. The dashed line indicates the cutoff value 20 used in hotspot selection. This cutoff corresponds to the p-value 1e-15 and 8e-22 for negative and positive LA results respectively.

Using a p-value cutoff 1e-5, we obtain 605 and 1213 gene pairs with positive/negative LA scores respectively. The locations of the linked marker blocks for these 1818 gene pairs are provided in Figure 4. By a stringent cutoff of at least 20 linkages per marker block, we identify 6 hotspots from positive LA results and 10 hotspots from negative LA results [see Table S6 of Additional file 2]. We compare the locations of these 2D-trait linkage hotspots with the 8 hotspots identified by 1D-trait mapping by Brem et al. [1]. Only one of the 16 hotspots is close (distance < 20 kb) to the eight 1D-trait mapping hotspots. It occurs at the 10-th hotspot (linked to 27 gene pairs) with negative LA scores. This hotspot is located at chromosome XV: 180 kb, which is 10 kb apart from the 8th 1D-hotspot (linked to 19 genes), which is located at chromosome XV: 170 kb, reported in Brem et al. [1]. However, there is no overlap between the 19 genes identified by 1D-trait mapping and any gene in the 27 gene pairs identified by 2D-trait mapping [see section C of Additional file 2].

Next, we ask the question whether some of these 1818 LA 2D-trait mapping results can also be obtained by 1D-trait mapping. Specifically, each 2D-trait mapping result is a triplet (*X*, *Y*, *Z*) where *X *and *Y *are two genes and *Z *is one marker block. In order for 1D mapping to link *X *to *Z *and *Y *to *Z*, the absolute value of correlation, |corr(*X*, *Z*)| and |corr(*Y*, *Z*)|, must be large enough. However, this is not the case. In fact, 95 percents of these values are less than 0.24. As a comparison, 95 percents of the 1D linkages mapped to eight 1D-hotspots of Brem et al. have correlation 0.59 or higher [see section D of Additional file 2]. An alternative way to temper the impact of chance errors is to control the number of gene pairs in each study. We shall restrict to functionally associated gene pairs next. Note that the reported FDRs hereafter are computed under the conservative assumption that all cases are null.

### Dynamic Co-expression Pattern of Gene Pairs within One Pathway

The pathway information is downloaded from Saccharomyces Genome Database [23]. Among the 139 pathways annotated by SGD, 121 of them include at least 2 genes with expression profiles in the yeast eQTL dataset [1]. The majority of these 121 pathways (78/121) have no more than five genes. Only 11 pathways have more than ten genes [Figure S2 in Additional file 1]. There are altogether 1711 gene pairs that can be formed from genes within the same pathway. The permutation p-value of the most positive or the most negative LA score for each gene pair is calculated based on 5000 permutations. At the permutation p-value cuto3 0.005, 207 gene pairs with positive LA scores (FDR = 4.13%) and 176 gene pairs with negative LA scores (FDR = 4.86%) are found^1^. Because of an overlap of 34 gene pairs, in total we get 349 unique gene pairs, covering 70 pathways [Figure S3 in Additional file 1]. The full list of these gene pairs and LA results are available at LA website [24].

We shall report the results of four pathways next. The first case is the leucine biosynthesis pathway. Brem et al. [1] have reported that expression levels of several leucine biosynthesis genes are linked to *LEU2 *locus due to the deletion of *LEU2 *in RM strain. We discuss how the dynamic co-expression of the leucine biosynthesis genes complements their findings. The second case is the *IMD3 *locus that mediates the co-expression of gene pairs from both histidine and purine biosynthesis pathways. The third and the fourth cases concern the phospholipid and purine biosynthesis pathways. The role of transcription factors will be discussed. To save space, the discussions of these two cases are given in section 2 of Additional file 1.

#### Leucine Biosynthesis Mediated by LEU2 Locus

**Figure 5:**
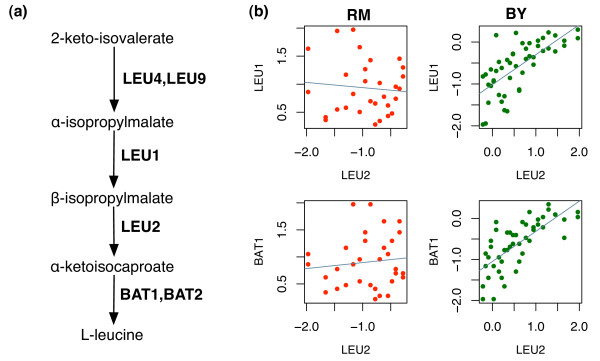
**2D mapping in Leucine biosynthesis pathway**. (a) Leucine biosynthesis pathway. (b) Co-expression pattern of (*LEU2*, *LEU1*) and (*LEU2*, *BAT1*) are mediated by genotype of marker block 75, to which *LEU2 *is cis-linked. Each green (red) dot indicates a yeast segregant of which *LEU2 *is inherited from BY (RM) strain.

There are six genes in this pathway. According to [1], RM strain has a null mutation in *LEU2*. Consistent with this information, the 1D-trait mapping shows that LEU2 is cis-linked and four of five other leucine biosynthesis genes *LEU1*, *LEU4*, *LEU9*, and *BAT1 *are trans-linked to *LEU2 *locus. All these trans-linked genes have elevated expression in RM strain, presumably compensating the loss of LEU2 in RM strain. Because of the negative shift in the mean, the overall correlations are found to be negative between LEU2 and the five other trans-linked leucine biosynthesis genes. With 2D-trait mapping, we find that the co-expression patterns between them and *LEU2 *also change as the genotype of *LEU2 *changes (Figure 5, Table 1). *LEU1 *and *BAT1*, two enzymes that catalyze the two steps in leucine biosynthesis pathway adjacent to where *LEU2 *works, have significant LA linkage (p-value < 0.0002). For segregants inheriting the normal *LEU2 *allele (BY allele) we can see the clear coexpression patterns between *LEU1 *and *LEU2 *and between *BAT1 *and *LEU2*. But for segregants with null *LEU2 *allele (RM allele), as expected, no significant co-expression patterns can be found. Weaker trans-linkage of *LEU4*, *LEU9 *to *LEU2 *locus and weaker dynamic co-expression pattern between *LEU4*, *LEU9 *and *LEU2 *are also observed. *LEU4 *and *LEU9 *are separated from *LEU2 *by *LEU1 *in the pathway.

**Table 1:** Co-expression of leucine biosynthesis genes mediated by the eQTL of *LEU2 *(marker block 75).

Gene1	Gene2	LA score	Corr(Gene1, Gene2)		
			
			Overall	RM	BY
*LEU2*	*LEU1*	0.365 **	-0.48	-0.10	0.70
*LEU2*	*BAT1*	0.335 **	-0.46	0.11	0.73
*LEU2*	*LEU9*	0.258 *	-0.42	0.11	0.44

The significant codes of LA scores are: '**' p ≤ 0.005, '*' 0.005 < p ≤ 0.01, '.' 0.01 < p ≤ 0.05. Same significant codes of LA scores are used for other tables.

#### Purine and Histidine Biosynthesis Mediated by IMD3 Locus

**Figure 6:**
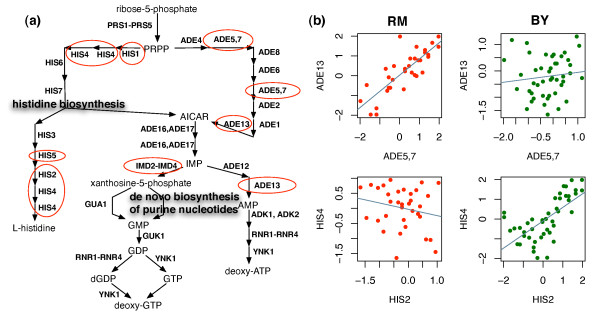
**2D mapping in histidine/purine biosynthesis pathway**. (a) Pathway of histidine biosynthesis and purine biosynthesis. (b) Co-expression pattern of (*ADE5*,*7*, *ADE13*) and (*HIS2*, *HIS4*) are mediated by genotype of marker block 473.

The two pathways, de novo biosynthesis of purine nucleotides and histidine biosynthesis, are connected and they are parts of super-pathway of histidine, purine, and pyrimidine biosynthesis (Figure 6(a)). With 2D-trait mapping, we find that co-expression patterns of two histidine biosynthesis genes (*HIS2*, *HIS4*) and two purine biosynthesis genes (*ADE5*,*7*, *ADE13*) are significantly linked to a locus where gene *IMD3 *is located (See Figure 6(b) and Table 2). *IMD3 *is one of the four IMP dehydrogenases (*IMD1*-*IMD4*) in yeast genome that catalyze the rate-limiting step in biosynthesis of purine [25]. When *IMD3 *locus is inherited from RM strain, we find strong positive correlation between the expression of *ADE5*,*7 *and *ADE13*, but negative correlation between the expression of *HIS2 *and *HIS4*. In contrast, when *IMD3 *locus is inherited from BY strain, We find strong positive correlation between the expression of *HIS2 *and *HIS4*, but the correlation between the expression of *ADE5*,*7 *and *ADE13 *drop to near zero (Table 2). In fact, if *IMD3 *locus is inherited from BY strain, overall, the genes in histidine biosynthesis pathway are more coherently co-expressed^2^. On the other hand, if *IMD3 *locus is inherited from RM strain, overall, the genes in purine biosynthesis pathway are more coherently co-expressed^3^. These results imply that *IMD3 *plays a significant role in mediating the histidine biosynthesis and the purine biosynthesis.

**Table 2:** Co-expression of histidine and purine biosynthesis genes mediated by *IMD3 *locus (marker block 473)

Gene1	Gene2	LA score	Corr(Gene1, Gene2)		
			
			Overall	RM	BY
*HIS2*	*HIS4*	0.417 **	0.46	-0.21	0.67
*ADE5,7*	*ADE13*	-0.440 **	0.58	0.82	0.12
*ADE5,7*	*ADE1*	-0.354.	0.34	0.62	-0.04
*ADE1*	*ADE6*	-0.310.	0.31	0.60	-0.01
*IMD3*	*HIS1*	-0.407 **	-0.11	0.56	-0.53
*IMD3*	*HIS5*	-0.433.	0.16	0.64	-0.34

We further find that the co-expression patterns of two gene pairs (*HIS1*, *IMD3*) and (*HIS5*, *IMD3*) are also linked to *IMD3 *locus [Table S2, and Figure S4 in Additional file 1]. These liquid association results reflect well the dynamic connection between the histidine and purine biosynthesis pathways. This finding cannot be explained by 1D-mapping. The expression of histidine/purine biosynthesis genes have low correlation with the genotype profile of the *IMD3 *locus^4^.

The above two examples show that the marker block, which mediates the 2D-trait contains an enzyme within the same pathway. There are altogether 10 such cases (corresponding 8 distinct marker blocks) [see Table S2 of Additional file 1]. In section 2 of Additional file 1, we also describe a different situation where the mediating marker block contains a TF known to regulate at least one gene in the 2D-trait.

### 2D-trait mapping for cis-null/all-trans loci

In this section, we shall discuss a more complicated situation encountered in 1D-trait mapping when loci with only trans-linkages but no cis-linkages are detected. If there is a cis-linked gene in a locus, a straightforward explanation of the trans-linkages is that the sequence polymorphism in the eQTL affects the expression of the cis-linked gene first, and then the cis-linked gene affects expression of the trans-linked genes. In this situation, we would expect to observe the overall co-expression between the cis-linked gene and the trans-linked genes. On the other hand, there are eQTL that do not harbor any cis-linked genes. This leads to a puzzling situation of where to find the likely local causative genes.

With 1D-trait mapping, we find altogether 76 genes trans-linked to the marker blocks that contain no cis-linked genes (see section 3 of Additional file 1). We further find out that there are only 7 marker blocks harboring at least 3 trans-linked genes but no cis-linked genes. We study the function of these trans-linked genes by GO Term Finder of SGD [26]. Among the 7 marker blocks, we find 3 of them have enriched GO term annotations:

1. Marker block 391: 8 trans-linked genes, enriched GO term "ATP metabolic process" (4 of 8 genes, p-value = 1.97e-7).

2. Marker block 335: 3 trans-linked genes, enriched GO term "formate metabolic process" (3 of 3 genes, p-value = 3.87e-10).

3. Marker block 446: 4 trans-linked genes, enriched GO term "mitochondrial electron transport, ubiquinol to cytochrome c" (2 of 4 genes, p-value = 0.00041).

We shall investigate the first case in detail and leave the discussion of the other two cases in section 3 of Additional file 1.

**Figure 7:**
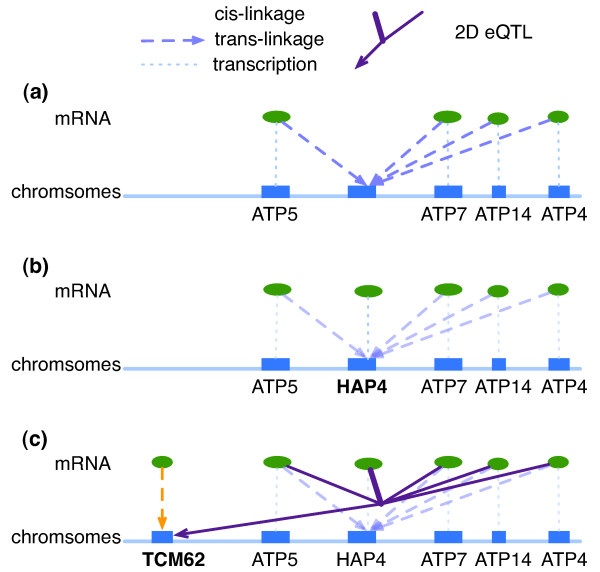
**Identification of *TCM62 *as the mediator of the co-expression patterns between *HAP4 *and its target genes**. (a) Expression level of *ATP4*, *ATP5*, *ATP7*, and *ATP14 *are trans-linked to chromosome XI: 235002 to 252763. (We skip *MEF2 *and *PPA2 *to simplify the graph) (b) We find that *HAP4 *is located in this eQTL region, but it is not cis-linked. (c) Co-expression pattern between *HAP4 *and those ATP genes are linked to *TCM62 *locus.

First, by 1D-trait mapping, eight genes functioning in ATP metabolism and aerobic respiration are linked to Chromosome XI: 235.0 kb to 252.8 kb (marker block 390–391) (Table 3, Figure 7(a)). *HAP4*, which encodes a transcription activator of respiratory genes [27], is found in this locus (Figure 7(b)).

**Table 3:** Eight genes that are trans-linked to marker block 390-39

Gene symbol	Description
*ATP4*	Subunit b of the stator stalk of mitochondrial F1F0 ATP synthase
*ATP5*	Subunit 5 of the stator stalk of mitochondrial F1F0 ATP synthase
*ATP7*	Subunit d of the stator stalk of mitochondrial F1F0 ATP synthase
*ATP14*	Subunit h of the F0 sector of mitochondrial F1F0 ATP synthase
*MEF2*	Mitochondrial elongation factor G-like protein
PPA2	Mitochondrial inorganic pyrophosphatase, required for mitochondrial function and possibly involved in energy generation from inorganic pyrophosphate
*CKS1*	Subunit of the Cdc28 protein kinase
*YCR102W-A*	Similar to several yeast probable membrane proteins, including *YNR075W *and *YFL062W*

Genome-wide TF binding data shows that Hap4 binds the upstream regions of *ATP5*, *ATP7*, and *ATP14 *[28]. However, *HAP4 *is not cis-linked since this locus is a cis-null/all-trans linkage spot. Consequentially, the correlations in expressions between *HAP4 *and any of the 8 trans-linked genes are low (from 0.02 to 0.38, with median 0.21).

To identify the possible dynamic co-expression patterns between *HAP4 *and the eight trans-linked genes, we take the expression profile of each of the eight trans-linked genes as *X*, the expression profile of *HAP4 *as *Y*, and the genotypes of all the 667 marker blocks as *Z *to calculate LA scores. We look for marker blocks appearing multiple times in the short list of marker blocks with best LA scores (20 most positive and 20 most negative). We find one marker block, marker block 41 (Chromosome II: 328.5 kb to 334.0 kb), appears six times (Table 4) as one of the marker block among the 20 marker blocks with most negative LA scores. We further find out that *HAP4 *co-expresses well with these genes if the sequence of marker block 41 is inherited from RM strain. The six LA scores listed at Table 4 show only a modest significance individually, but collectively they are significant with p-value of 0.0064. More specifically, we ask whether it is possible that a permuted marker block can have more extreme LA scores than marker block 41 at all the six cases. Out of 5000 permutations, we find only 32 such cases, yielding a permutation p-value of 0.0064. Using 1D-trait mapping, a gene, *TCM62*, is found to be cis-linked to this marker block (Figure 7(c)). It is known that Tcm62 forms a complex containing at least three SDH subunits Sdh1, Sdh2 and Sdh3 [29], and all these SDH genes are involved in aerobic respiration [30], which is consistent with the function of *HAP4 *and its target genes. Thus marker block 41, or more specifically, gene *TCM62 *is a plausible candidate that mediates the co-expression pattern between *HAP4 *and its target genes.

**Table 4:** Co-expression between *HAP4 *and its six target genes are mediated by a locus in marker block 41.

Gene1	Gene2	LA score I	Corr(Gene1, Gene2)			LA
				
			Overall	RM	BY	score II
*HAP4*	*ATP4*	-0.264	0.38	0.60	0.10	0.321
*HAP4*	*ATP5*	-0.231	0.29	0.51	0.04	0.237
*HAP4*	*ATP7*	-0.216	0.25	0.49	0.01	0.292
*HAP4*	*ATP14*	-0.292	0.20	0.50	-0.16	0.367
*HAP4*	*MEF2*	-0.256	0.21	0.53	-0.10	0.381
*HAP4*	*PPA2*	-0.334	0.21	0.57	-0.19	0.322

For the column of LA score I, we use Z = marker profile of Marker block 41. We ask whether more extreme LA scores can be observed for all the six cases by chance. Using random permutation, we get 32 positive results from 5000 permutations, corresponding to a permutation p-value 0.0064. For the column of LA score II, we use Z = expression profile of *TCM62*. We ask whether more extreme LA scores can be observed for all the six cases by chance. Using random permutation, we get 10 positive results from 5000 permutations, corresponding to a permutation p-value 0.002.

## Discussion

The goal of eQTL studies is to map complex traits to genetic loci with the aid of gene expression data. Since a single gene/protein is unlikely to affect a complex trait by itself, it would be more informative to take higher order cellular organization into consideration. The co-expression pattern of two genes may reflect the status of the regulatory mechanism. If the majority of gene pairs (or gene pairs from the rate-limiting steps) in a pathway show coherent patterns of expression, we would expect the pathway to function more effectively. Our approach of 2D-trait mapping is a novel way to connect the genetic variation with higher order biological modules via gene expression profiles.

It is important to bear in mind, however, that a significant score *LA*(*X*, *Y*|*Z*) does not necessarily implies that there must be a direct causal relationship of "*Z *affects *X *and *Z *affects *Y *". It is feasible for *Z *to affect *X *only, but *X *and *Y *are correlated through other unspecified factors, thereby changing the conditional correlation. There are many factors, including environment, epigenetics, signaling molecules, microRNA, etc., which may have more direct influence on the correlation between *X *and *Y*. Our method should be viewed as complementary to the more traditional and multivariate QTL analysis, but certainly not as the replacement.

We have illustrated how the standard 1D-trait mapping and the 2D-trait mapping can complement each other to broaden the scope of eQTL studies. The similarity between the binary LA scoring method and the continuous LA scoring method offers an additional advantage. Let's reconsider how the locus of *HAP4 *and the locus of *TCM62 *mediate the ATP metabolism and aerobic respiration. An alternative analysis can begin with using 6299 gene expression profiles as Z to find genes with highest LA score, *LA*(*X*, *Y*|*Z*), where *X *= the expression profile of each of the 8 trans-linked genes, and *Y *= *HAP4 *expression profile. We find that *TCM62 *appears six times as one of the top 20 genes with highest LA scores. Combined with 1D-trait mapping result of cis-linkage for *TCM62*, we may propose a possible scenario that the DNA polymorphism in marker block 41 affects the expression of *TCM62*, which in turn affects the co-expression pattern of *HAP4 *and its target genes (Table 4).

Although we choose to study the dynamic co-expression patterns of genes belonging to one metabolic pathway, other functional categories such as gene ontology terms or protein complexes can also be employed.

Our 2D-trait mapping can be generalized in two directions. The first direction is to extend the method to the co-expression of more than 2 genes. The method of projective LA [31] should be applicable here. On the other hand, for 1D-trait mapping, one gene expression profile can be mapped to more than one locus. How to model and detect interactions between loci (for 1D-trait mapping) has received great attention recently. Likewise, we should allow 2D-trait to be mapped to more than one locus. The dimensionality issue is surely to escalate further if high dimensional traits with multi-loci are considered systematically in a comprehensive manner.

Another possibility of conducting the 2D mapping is to use the direct difference of Pearson correlation coefficients (denoted by DCC):

(3) $$\begin{array}{lll} \text{DCC} & \text{=} & \left[E(X-E(X|Z=1))(Y-E(Y|Z=1))]/SD(X|Z=1)SD(Y|Z=1\right)\\ & - & [E(X-E(X|Z=0))(Y-E(Y|Z=0))]/SD(X|Z=0)SD(Y|Z=0 \end{array}$$

where *SD *stands for standard deviation. For the protocol case as depicted by the schematic Figure 1(b), the two measures are equivalent because *E*(*X*|*Z *= 1) = *E*(*X*|*Z *= 0), *E*(*Y*|*Z *= 1) = *E*(*Y*|*Z *= 0), *SD*(*X*|*Z *= 1) = *SD*(*X*|*Z *= 0), and *SD*(*Y*|*Z *= 1) = *SD*(*Y*|*Z *= 0). If DCC is used for other situations, then one must be aware of the different biological interpretation of what it means by co-expression/co-regression of two genes. This is because (i) two different baseline expressions are now used in defining up-regulation or down-regulation of a gene and (ii) two different scales are used in defining the strength of up-regulation or down-regulation of a gene. In contrast, LA method uses only one common baseline and only one common scale. Another difference between LA and DCC is that while LA can be applied to both discrete and continuous Z, it is not easy to obtain an implementable version of DCC for a continuous Z.

## Abbreviations

eQTL: gene expression quantitative trait loci; LA: liquid association; FDR: false discovery rate; TF: transcription factor.

## Authors' contributions

All three authors designed the study, introduced concepts and methods, analyzed data, interpreted the findings and wrote up the paper. WS and SY conducted the enabling computation for the pathway study and the full genome study respectively.

## Note

^1^The FDR is about 2% and 8% if we use p-value cutoff 0.001 or 0.01 respectively

^2^We observe overall higher correlation for the 21 gene pairs in histidine biosynthesis pathway if *IMD3 *is inherited from BY strain. We compare the correlations by paired t-test and Kolmogorov-Smirnov test using R functions t.test and ks.test respectively. The two-sided p-value of paired t-test is 3.8*e*^-2 ^and for Kolmogorov-Smirnov test is 5.4*e*^-3^

^3^We observe overall higher correlation for the 231 gene pairs in purine biosynthesis pathway if *IMD3 *is inherited from RM strain. We compare the correlations by paired t-test and Kolmogorov-Smirnov test using R functions t.test and ks.test respectively: The two-sided p-value of paired t-test is 2.4*e*^-10 ^and for Kolmogorov-Smirnov test is 1.3*e*^-8^

^4^Correlations between 7 histidine biosynthesis genes and genotype profile of the IMD3 locus vary from -0.27 to 0.23, with median 0.03. The correlations for 22 purine biosynthesis genes vary -0.34 to 0.09, with median -0.18

## Supplementary Material

Additional file 1 **Supplementary Materials, Part I**. Supplementary description/results of marker block construction and pathway studies. Click here for file

Additional file 2 **Supplementary Materials, Part II**. Supplementary genome-wide results of 2D-trait mapping. Click here for file
