# Supplementary material for: Trait-trait dynamic interaction: 2D-trait eQTL mapping for genetic variation study
Source: BMC Genomics. 2008 May 23;9:242. doi: 10.1186/1471-2164-9-242 (PMC2432080; doi:10.1186/1471-2164-9-242)
Supplement: Additional file 1 — Supplementary Materials, Part I. Supplementary description/results of marker block construction and pathway studies. [file 1471-2164-9-242-S1.pdf]

Supplementary Materials, Part I for "trait-trait dynamic interaction: 2D-trait eQTL mapping for genetic variation study"

Wei Sun, Shinsheng Yuan and Ker-Chau Li

May 12, 2008

1 Construct Marker Blocks

Brem et al. [1] identified 3313 genetic markers in 40 yeast segregants. These segregants are from a cross of two parent strains RM and BY. In each segregant, the genotype of one marker can be either 0 (RM) or 1 (BY) depending on from which parent strain the sequence polymorphism is inherited. If the origin is ambiguous, it will be set as missing value. Thus each marker corresponds to a vector of 0, 1 or missing value. Many neighboring markers are similar because they are too close to allow recombination. To reduce the computation burden and simplify the result, we combine all the 3313 markers into 667 marker blocks. The following flowchart (Figure 1) shows the procedure of generating marker blocks.

We include a marker into a marker block if the Manhattan distance between this marker and any marker in the marker block is no greater than 1.25. If there is no missing value, Manhattan distance between two vectors $X = (x_1, x_2, ..., x_n)$ and $Y = (y_1, y_2, ..., y_n)$ is $\sum_{i=1}^{n} |x_i - y_i|$. However, if either x_i or y_i is missing, the term $|x_i - y_i|$ is excluded from the summation. If altogether m terms are excluded due to missing values, the summation is adjusted by multiplying $n/(n-m)$. Thus Manhattan distance between two markers M_i and $M_j \leqslant 1.25$ means that: (1) M_i and M_j are the same; or (2) M_i and M_j are different at one segregant and more than 80% of the segregants have non-missing genotypes in both M_i and M_j. If we require all the markers within one marker block to be the same in non-missing values (Manhattan distance = 0), we will end up with 1027 marker blocks and many of them are different at only one segregant. If we increase the cut-off value, the number of marker blocks decreases (Table 1). We do not want to sacrifice too much information in order to get less marker blocks, so we choose the cutoff 1.25.

At the end, we dichotomize the marker block consensuses into binary values. There are altogether 480 missing values (1.8%) in the 667 marker block profiles. Among them, 173 values are missing because the consensus is between 0.25 and 0.75; the other 307 values are missing because the genotype is missing for all the markers in the corresponding marker block. Our marker block construction method is a greedy algorithm because we add as many markers as possible into the current marker block. Under the criterion that any two

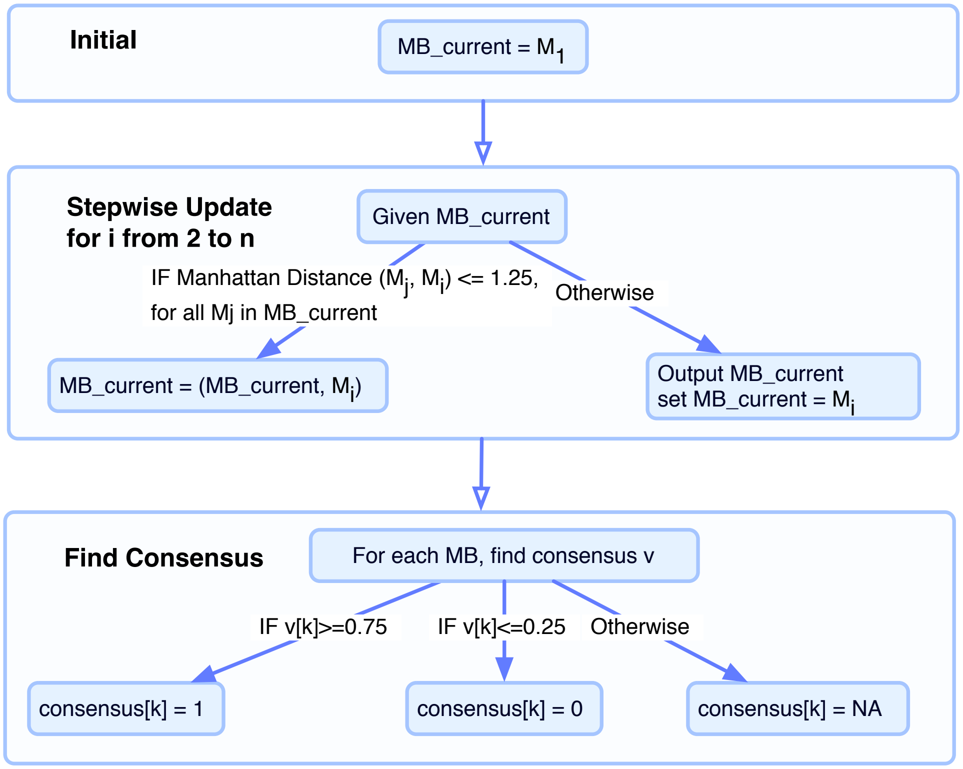

Figure 1: The procedure of constructing marker blocks

We construct marker blocks chromosome by chromosome. Suppose there are n markers in one chromosome, ordered by genomic location and named as M_1, M_2, ..., M_n. We define MB_current as the marker block that is currently under construction. In the initial step, let the MB_current be the first marker (M_1) in the chromosome. During stepwise update, for i from 2 to n, we decide whether to combine maker M_i into the current maker block or to finish the current marker block and set M_i as the new MB_current. At the end, we get all the marker blocks at this chromosome, and then find out the consensus of each marker block. The consensus is then dichotomized. It is set to be 0 if no less than 75% of the markers in this marker block equals to 0; it is set to be 1 if no less than 75% of the markers in this marker block equals to 1. Otherwise it is set as NA.

Table 1: Number of marker blocks corresponding to different distance cutoffs

Number of marker blocks (Counts) corresponding to different cutoffs of Manhattan distance (Distance). Actually, any distance cutoff between 1.25 and 1.95 does not make much difference. When the cut-off becomes 2.0, it begins to allow two markers within one marker block to be different at two segregants, so the number of marker blocks drops significantly.

Distance	1	1.1	1.2	1.25	1.3-1.5	1.6-1.9	2.0	2.1	2.2	2.3	2.4	2.5
Counts	854	731	680	667	666	665	603	555	524	501	492	484

markers within one marker block have a distance no greater than a given constant, our method will result in fewest marker blocks.

2 Dynamic Co-expression of Gene Pairs Within One Pathway

Table 2: A subset of LA results for gene pairs from one pathway

This table shows a subset of significant LA results $LA(X, Y|Z)$ such that one gene Z_G in the marker block (Z) is in the same pathway as the gene pair X and Y.

X	Y	Marker Block	Z_G	Pathway
DUT1	CDC21	64	DUT1	de novo biosynthesis of pyrimidine deoxyribonucleotides
ADE5,7	ADE13	473	IMD3	de novo biosynthesis of purine nucleotides
PAN6	YKL088W	396	YKL088W	pantothenate and coenzyme A biosynthesis
LEU2	LEU1	75	LEU2	leucine biosynthesis
LEU2	BAT1	75	LEU2	leucine biosynthesis
PRS2	PRS1	385	PRS1	superpathway of histidine, purine, and pyrimidine biosynthesis
ADE5,7	ADE12	238	ADE6	de novo biosynthesis of purine nucleotides
URA8	URA6	372	URA8	de novo biosynthesis of pyrimidine ribonucleotides
SDH1	ACO1	654	CIT3	TCA cycle, aerobic respiration
SDH1	FUM1	654	CIT3	TCA cycle, aerobic respiration

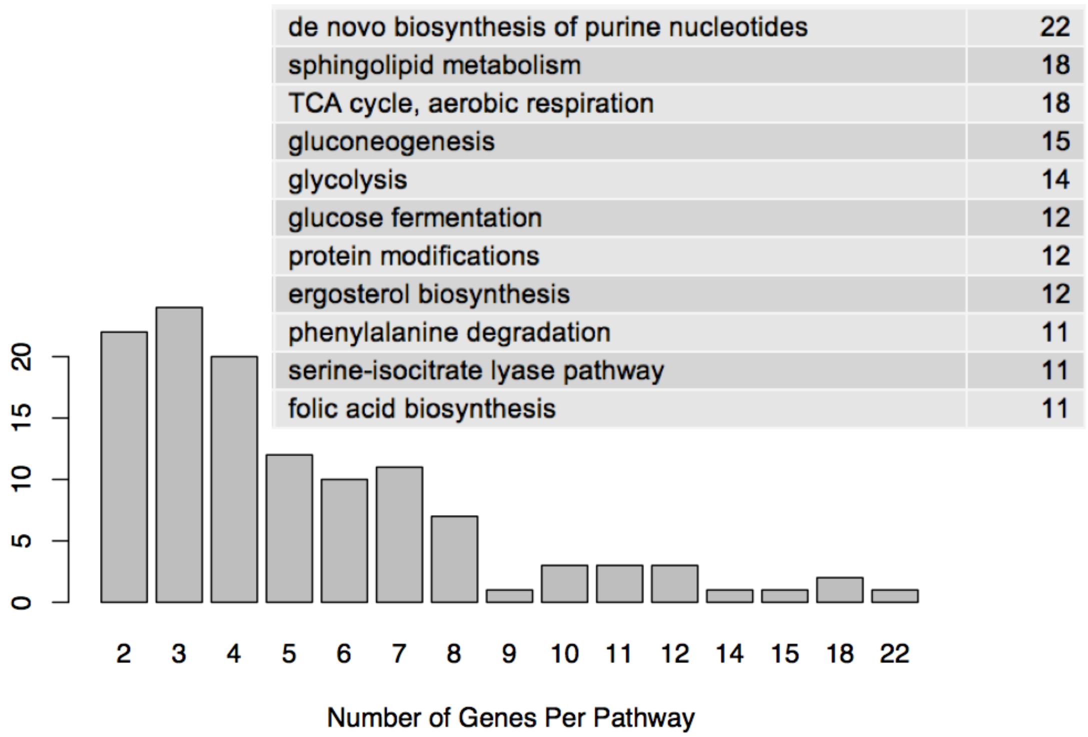

de novo biosynthesis of purine nucleotides	22
sphingolipid metabolism	18
TCA cycle, aerobic respiration	18
gluconeogenesis	15
glycolysis	14
glucose fermentation	12
protein modifications	12
ergosterol biosynthesis	12
phenylalanine degradation	11
serine-isocitrate lyase pathway	11
folic acid biosynthesis	11

Figure 2: Pathway information

The bar-plot shows number of genes per pathway for the 121 pathways we used. Most pathways include no more than 10 genes. The 11 pathways that include more than 10 genes are also listed.

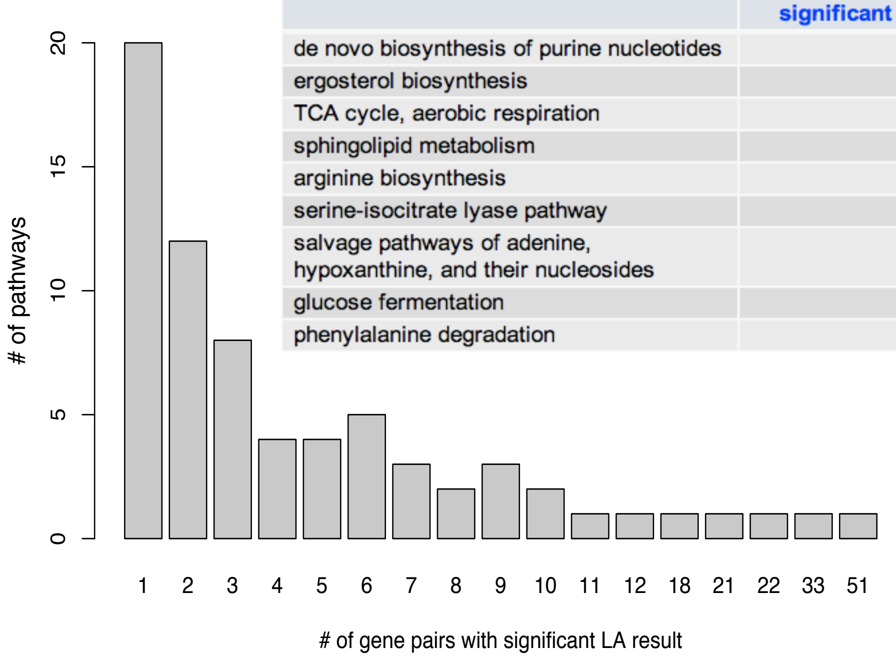

pathway	# of gene paris with significant LA scores	# of genes
de novo biosynthesis of purine nucleotides	51	22
ergosterol biosynthesis	33	12
TCA cycle, aerobic respiration	22	18
sphingolipid metabolism	21	18
arginine biosynthesis	18	7
serine-isocitrate lyase pathway	12	11
salvage pathways of adenine, hypoxanthine, and their nucleosides	11	8
glucose fermentation	10	12
phenylalanine degradation	10	11

Figure 3: Distribution of significant LA result in different pathways
There are altogether 70 pathways with at least one significant LA result. This bar-plot shows the distribution of the number of genes pairs with significant LA scores in these 70 pathways. The 10 pathways having more than 10 genes pairs with significant LA scores are also listed. For example, the first row in the table is "de novo biosynthesis of purine nucleotide 51 22", which means there are 22 genes in this pathway, and 51 gene pairs from the 22 genes have significant LA scores.

Figure 4: Co-expression pattern of (HIS1, IMD3) and (HIS5, IMD3) are mediated by genotype of marker block 473, where IMD3 is located.

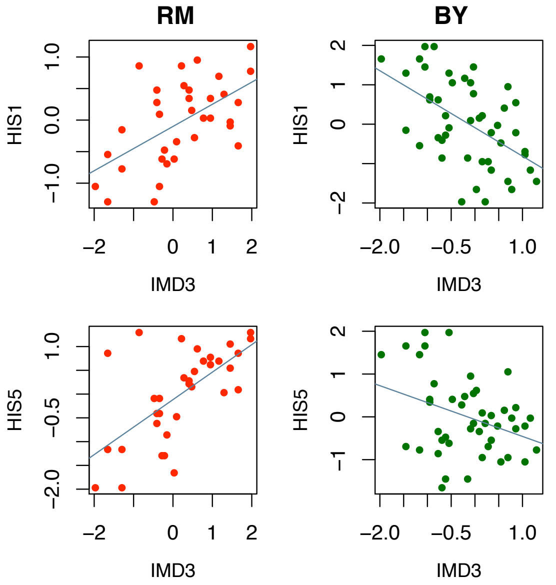

Transcription factor Ino2 and Phospholipid Biosynthesis

In the phospholipid biosynthesis pathway, our 2D-trait mapping results reveal two gene pairs (PSD2, CHO1) and (PSD2, CDS1) significantly linked to INO2 locus (Figure 5, Table 3). INO2 does not encode an enzyme in this metabolic pathway. However, its gene product forms a basic helix-loop-helix transcription activator with Ino4. This transcription activator regulates phospholipid biosynthetic genes [2]. Table 3 shows that PSD2 strongly co-expresses with CDS1 and CHO1 if INO2 locus is inherited from BY strain. Otherwise the correlations between the expression of PSD2 and the expression of CDS1 and CHO1 are negative. Again, 1D-mapping cannot identify the INO2 locus. The correlation between CDS1, CHO1, PSD2 and the genotype profile of INO2 locus are weak: 0.00, -0.24, and -0.25 respectively.

Transcription factor Crt10 and Purine Biosynthesis

We return to the purine biosynthesis. The 2D-trait mapping also find the linkages of (IMD2, RNR3) and (IMD3, RNR3) to a locus where CRT10 is located. (Figure 6, Table 4). By 1D-trait mapping, CRT10 is cis-linked to this locus. In a recent study, Fu et al. [3] showed that Crt10 is a transcription factor, which regulates the expression of RNR2 and RNR3. In fact, expressions of (IMD2, IMD3, GUA1) have strong positive correlations with expressions of (RNR2, RNR3, RNR4) if and only if the CRT10 locus is inherited from

Figure 5: (a) Phospholipid biosynthesis pathway (b) Co-expression pattern of (PSD2, CDS1) and (PSD2, CHO1) are mediated by genotype of marker block 113, where INO2 is located.

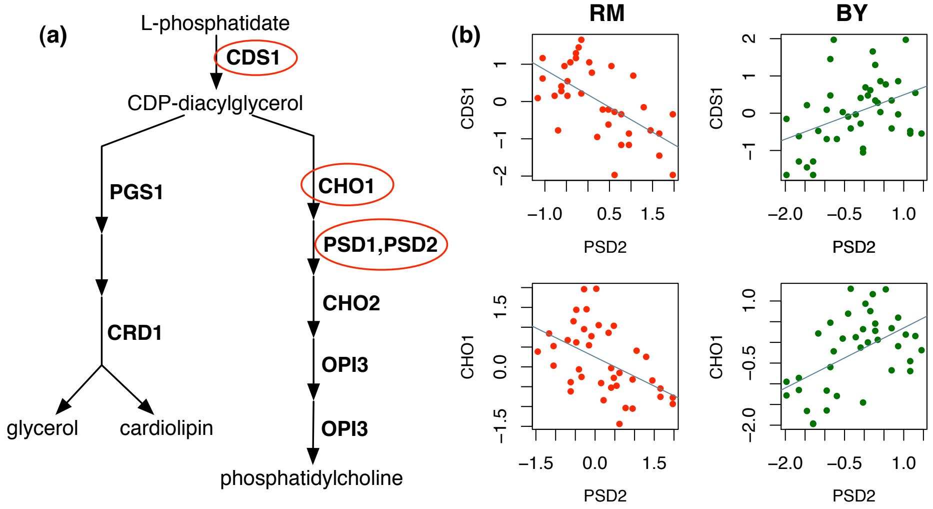

Table 3: Co-expression of phospholipid biosynthesis genes mediated by INO2 locus (marker block 113)

Gene1	Gene2	LA score	Corr(Gene1, Gene2) Overall	RM	BY
PSD2	CDS1	0.420 **	-0.06	-0.61	0.41
PSD2	CHO1	0.410 **	0.08	-0.50	0.51

BY strain. If it is inherited from RM strain, the correlations are either negative or close to zero (Table 4). The co-expression of RNR genes and IMD genes is important for purine biosynthesis since both IMD genes and RNR genes play important roles in this pathway. IMD genes catalyze the rate-limiting step in the biosynthesis of purine [4]. Those RNR genes belong to ribonucleotide-diphosphate reductase (RNR) complex, which catalyzes the rate-limiting step in dNTP synthesis [5]. Again, this 2D-mapping result cannot be found by 1D-mapping because the correlation between the expression of IMD and RNR genes and the genotype profile of the CRT10 locus is small (from -0.05 to 0.11).

Figure 6: Co-expression pattern of (RNR3, IMD2) and (RNR2, IMD2) are mediated by genotype of marker block 578, where CRT10 is cis-linked.

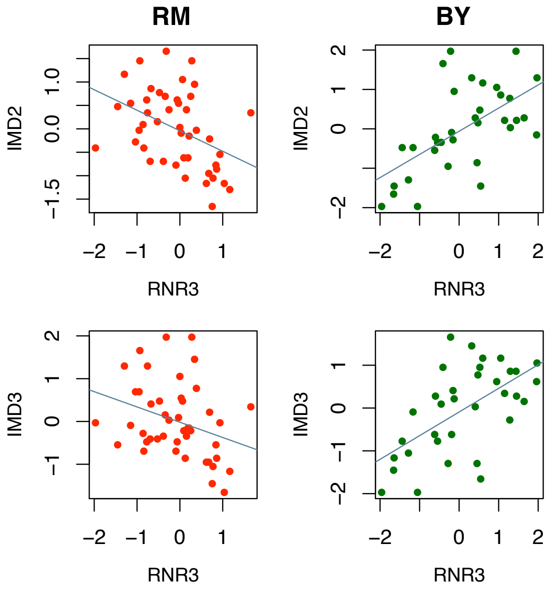

Table 4: Co-expression of purine biosynthesis genes mediated by CRT10 locus (marker block 578)

Gene1	Gene2	LA score	Corr(Gene1, Gene2)		
			Overall	RM	BY
IMD2	RNR2	0.341 .	0.41	0.08	0.65
IMD2	RNR3	0.469 **	0.17	-0.42	0.60
IMD3	RNR3	0.469 **	0.18	-0.32	0.59
IMD3	RNR4	0.328 **	0.37	0.04	0.65
GUA1	RNR2	0.379 *	0.44	0.08	0.70
GUA1	RNR3	0.353 *	0.07	-0.41	0.40
GUA1	RNR4	0.397 **	0.41	0.01	0.79

3 2D-trait Mapping for Cis-null/All-trans Loci

In order to identify Cis-null/All-trans loci, we need to first define the cis-linkage and trans-linkage explicitly. Use G to denote a gene and use M to denote a marker (or a marker block). Brem et al. [1] define a linkage from G to M as cis-linkage if the distance between G and M is smaller than 10 kb. We adopt the same definition. Student's t-test is used to quantify the linkage strength. Different p-value cutoffs are used to detect trans-linkage (5e-5) and cis-linkage (5e-4) because the multiple testing problem is much more serious for trans-linkage. We found altogether 76 genes that are only trans-linked to those marker blocks to which no gene is cis-linked (Figure 7). The list of these 76 genes can be found in website: http://www.stat.ucla.edu/∼sunwei/2D-eQTL.htm

We identify the spots that harbor eQTLs of at least 3 genes and cover at most 2 adjacent marker blocks for further analysis. Altogether 7 such spots are obtained, corresponding to a total of 44 trans-linked genes. For each spot, we measure the functional enrichment of the genes linked to it by GO term finder in SGD (http://db.yeastgenome.org/cgi-bin/GO/goTermFinder). The information of each spot is listed below:

Marker block 169-170 (9 genes) The trans-linked genes are: SKS1, YPR013C, YHL026C, RIM15, ECM34, YAP6, YHL042W, SOK1, and MKK1; no significantly enriched GO term is found

Marker block 335 (3 genes) The trans-linked genes are: FDH2, YPL276W, and FDH1. There is one significantly enriched GO term: formate metabolic process (3 of 3 genes, p-value = 3.87e-10). YJL206C is located in this linkage region (Marker block 335). YJL206C is a TF that regulates expression of FDH1, FDH2, and YPL276W. The co-expression patterns between YJL206C and FDH1, FDH2, YPL276W are linked to marker

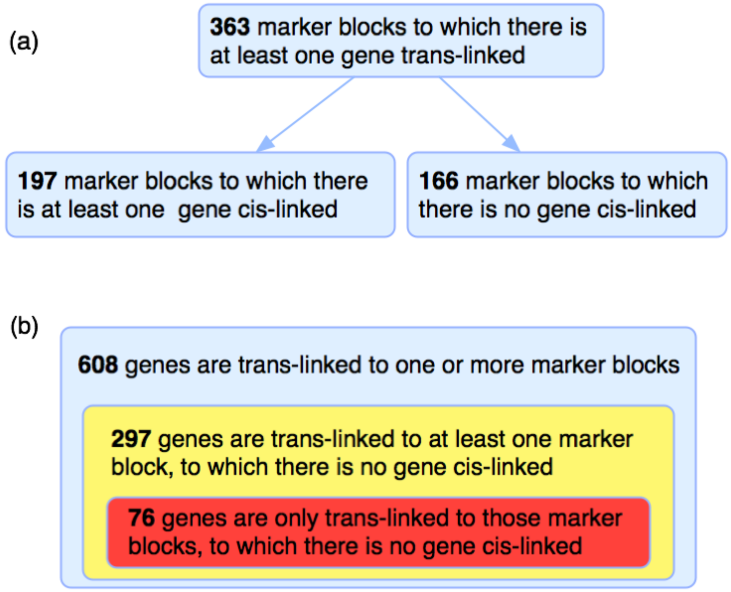

Figure 7: Identify Cis-null/All-trans marker blocks and corresponding genes
(a) Number of marker blocks that have trans-linked gene and the subset that have (or do not have) cis-linked gene. **(b)** Identification of the 76 genes that are trans-linked to one of cis-null/all trans loci.

block 388-392, where an eQTL of aerobic respiration genes is located. Figure 8 illustrates the dynamic co-expression pattern between YJL206C and FDH2.

Marker block 391 (8 genes) The trans-linked genes are: PPA2, ATP7, ATP4, YCR102W-A, ATP5, CKS1, MEF2, and ATP14. There is one significantly enriched GO term: ATP metabolic process (4 of 8 genes, p-value=1.97e-7). The 2D linkages in this hot spot is discussed in detail in main text.

Maker block 446 (4 genes) The trans-linked genes include COX15, CYT1, RIP1, and NDE1. There is one enriched GO term: mitochondrial electron transport, ubiquinol to cytochrome c (2 out of 4 genes, p-value=0.00041). It is known that Hap1 regulates expression levels of COX15 and CYT1. HAP1 harbors one eQTL hot spot (marker block 449). Brem et al. [1] suggested that HAP1 is the corresponding causal gene because the genes linked to it share HAP1 binding motifs, including some known target genes of Hap1. Besides, HAP1 is also cis-linked to marker block 449. It is possible that expression of COX15 and CYT1 are also affected by the HAP1 locus. However, due to some extra perturbation, they are linked to marker block 446. Figure 9 shows that the co-expression pattern

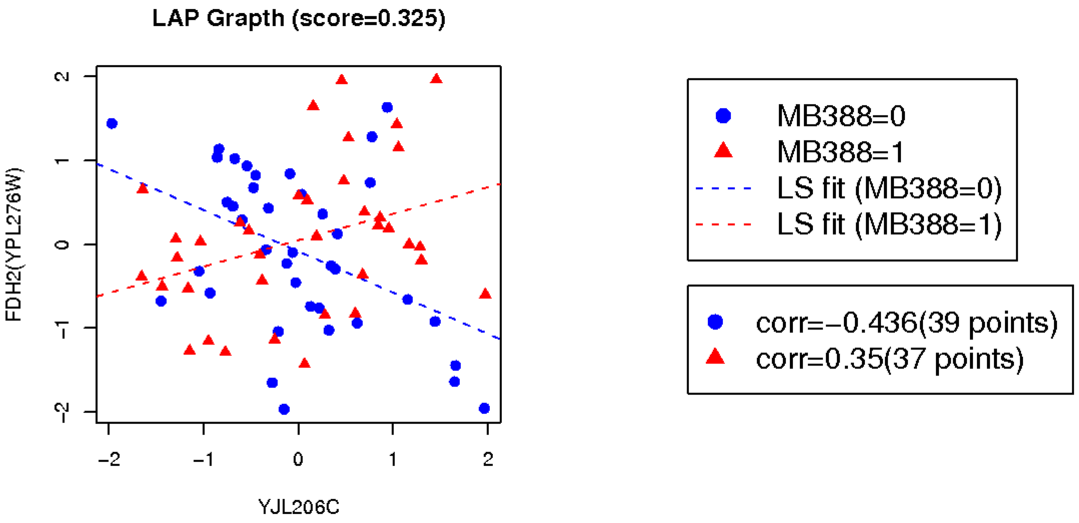

Figure 8: 2D linkage of YJL206C and FDH2

Dynamic co-expression pattern between YJL206C and FDH2 is mediated by marker block 388-392, where one group of genes related with aerobic respiration are trans-linked.

between HAP1 and CYT1 are linked to marker block 592, where CYT1 itself is located, similar 2D linkage can be observed for COX15. Thus one explanation is that the expression of CYT1 and COX15 are affected by both the sequence polymorphism of HAP1 and CYT1.

Maker block 471 (10 genes) The trans-linked genes include MPM1, YKR021W, FUN19, RNT1, PEX18, YLR143W, COX18, NOP7, YDR401W, and TOP2. There is no GO term significantly over-represented.

Maker block 569-570 (3 genes) The trans-linked genes are TYR1, PRP3, and RIT1. There is no GO term significantly over-represented.

Maker block 663-664 (7 genes) The trans-linked genes are POL12, GIN4, PIM1, CLU1, CDC9, TIM50, and TOF1. There is one enriched GO term: DNA-dependent DNA replication (3 of 7 genes, p-value=0.00851)

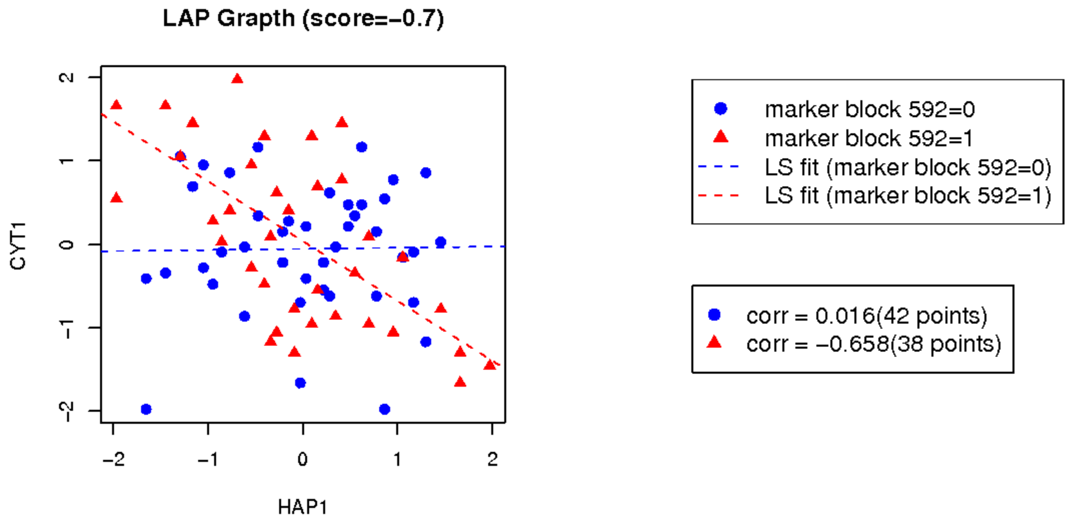

Figure 9: 2D linkage of YJL206C and FDH2

Dynamic co-expression pattern between HAP1 and CYT1 mediated by marker block 592, where CYT1 is located.

References

[1] Brem RB, Yvert G, Clinton R, Kruglyak L: **Genetic dissection of transcriptional regulation in budding yeast**. *Science* 2002, **296**(5568):752–755.

[2] Ambroziak J, Henry SA: **INO2 and INO4 gene products, positive regulators of phospholipid biosynthesis in Saccharomyces cerevisiae, form a complex that binds to the INO1 promoter**. *J Biol Chem* 1994, **269**(21):15344–15349.

[3] Fu Y, Xiao W: **Identification and characterization of CRT10 as a novel regulator of Saccharomyces cerevisiae ribonucleotide reductase genes**. *Nucleic Acids Res* 2006, **34**(6):1876–1883.

[4] Hyle JW, Shaw RJ, Reines D: **Functional distinctions between IMP dehydrogenase genes in providing mycophenolate resistance and guanine prototrophy to yeast**. *J Biol Chem* 2003, **278**(31):28470–28478.

[5] Yao R, Zhang Z, An X, Bucci B, Perlstein DL, Stubbe J, Huang M: **Subcellular localization of yeast ribonucleotide reductase regulated by the DNA replication and damage checkpoint pathways**. *Proc Natl Acad Sci U S A* 2003, **100**(11):6628–6633.
