# Supplementary material for: Trait-trait dynamic interaction: 2D-trait eQTL mapping for genetic variation study
Source: BMC Genomics. 2008 May 23;9:242. doi: 10.1186/1471-2164-9-242 (PMC2432080; doi:10.1186/1471-2164-9-242)

Supplementary Materials, Part II for ``trait-trait dynamic interaction: 2D-trait eQTL mapping for genetic variation study''

Section A: Genome-wide LA scores and significance assessment

The genome wide LA scores are computed for 16,463,269 gene pairs across the whole genome. For each gene pair, we search for the best marker blocks with positive or negative scores. After that, we have two sets of LA linkage scores, one for positive values and one for the negative values.

The null reference distribution is generated by the following procedure.
1. Randomly generate the values from the population of all expression values.
2. In this random drawing, we only draw forty values first and the rest of forty values are selected based on the previous random drawing results. For instance, if we randomly pick the expression value from 1000th row and 3rd column, the corresponding 1000th row and 43th column will be selected for preserving the dye-swap structure in the original data set.
3. After repeat step 1 and 2 for 1 million times, we obtain 1 million randomly generated gene pairs.
4. For each randomly generated gene pair, we compute the LA scores for all marker blocks and find its highest positive and negative marker blocks as well as the corresponding LA scores.

In the Figure 1, the qq-plot is shown for the genome wide LA linkage scores versus the reference LA linkage scores. As one can see that the apparent straight line is observed from both TOP (positive) and BOT (negative) qq-plots. In order to detect the subtle difference between the distributions, Figure 2 plots the difference between the quantiles of the two distributions against the quantile of the reference distribution. From Figure 2, we observe that the difference of these two distribution increases when the reference LA score gets larger. This shows that the higher LA linkage score is, the less likely it is resulted from random chance.

Figure 1: qq-plot of Genome Wide LA scores versus Random Generated LA scores.

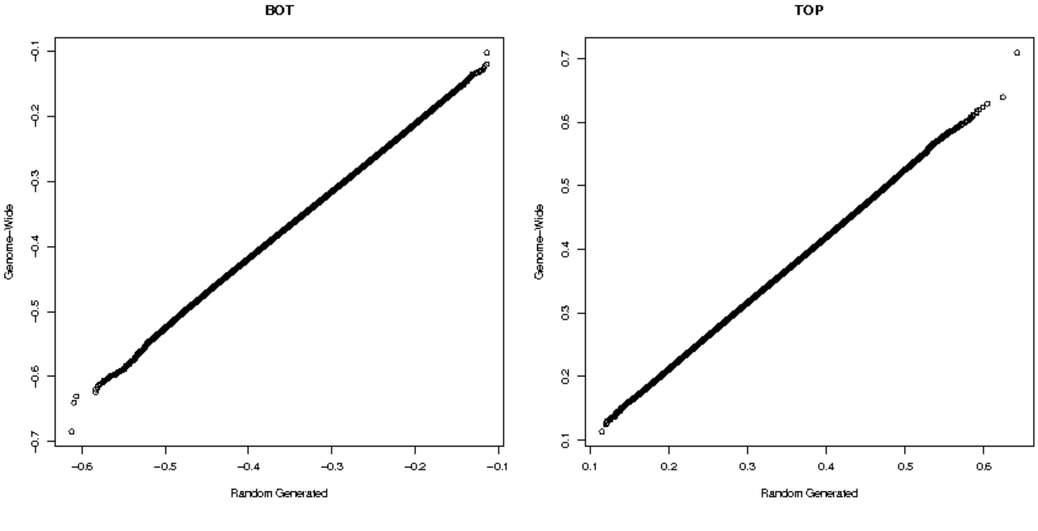

Figure 2: Quantile vs Difference in Quantile.
Abbreviations: RG, randomly generated LA scores; GW, Genome Wide LA scores

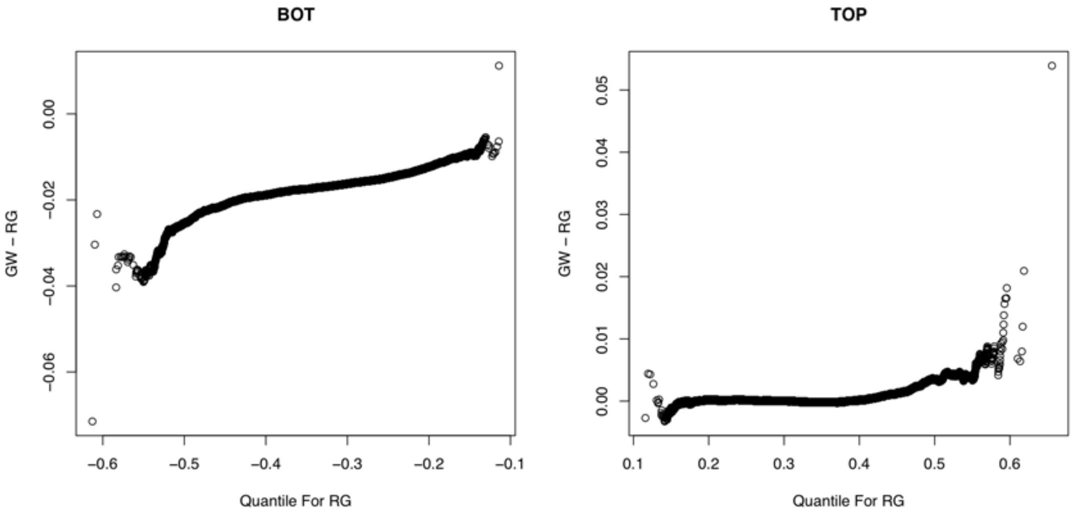

One simple way to estimate the FDR is to find out the ratio of expected number of LA scores from randomly generated sample and the observed number from genome wide distribution. In Figure 3, the relation between FDR and log(P-value) is plotted for both positive (TOP) and negative (BOT) LA scores. For each p-value, we find the corresponding cutoff of LA score and count the number of discoveries from genome wide LA scores, and then calculate FDR. For example, for p-value 1e-3, based on the reference distribution, the corresponding LA score cutoffs are -0.4867 (BOT) and 0.4575 (TOP). The corresponding number of discoveries are 49646 (BOT) and 47445 (TOP). This will give the estimated FDR 33.1% (BOT) and 34.7% (TOP).

Figure 3: P-value versus FDR.

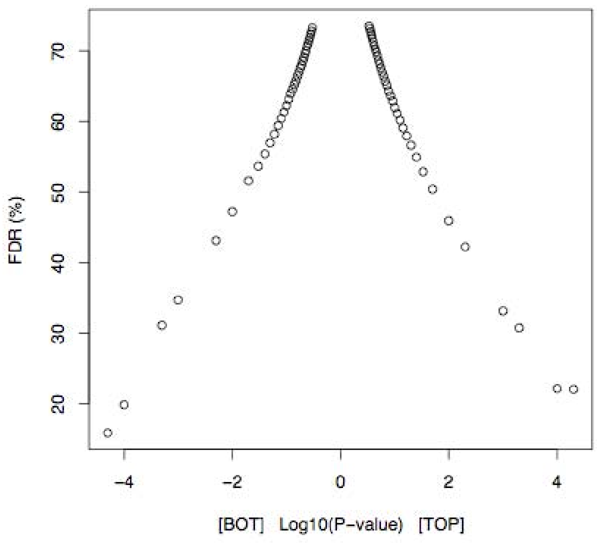

Our reference distribution could be regarded as a mixture of various distributions. The power of the test will be essentially reduced. For this genome wide study, we would like to demonstrate that the signal is essentially not that strong but still detectable. The high FDR is not that surprising since the majority of gene pairs are not expected to be related. This is why we also restrict our study on a subset of gene pairs based on pathway information.

Section B: Top/Bottom 20 LA 2D-trait Mapping Results from Genome-wide Study

In Table 1, we list the 20 gene pairs with the highest positive or negative LA scores from genome wide 2D-Linkage result. In Tables 2 and 3, gene pairs mapped to the overly represented marker block 227 are listed. Based on annotation from SGD (Saccharomyces Genome Database, http://www.yeastgenome.org/), *YGR146C* has no known function but is induced by transcription factor Aft2p. However, when we look at the annotations for its paired genes, we found that *KRR1, SAS10, RRB1, URB1, RPF1, LTV1, HTS1* are all involved or required in assembly of ribosome or ribosomal biogenesis (see Table 3 for the gene by gene annotations). Since *YGR146C* is still unknown, we look at the genes located within the marker block 227. We found *YGL101W*, *SEH1*, *LSG1*, *RUF2* and *USE1*. However, if we look at the gene located next to *YGL101W*, we found *RPL28*, whose gene expression profile has a correlation of 0.2240 with the marker profile of marker block 227. If we search for the genes, which trans-link to the marker block 227, we find that *MRM2*, Mitochondrial rRNA Methyl transferase, has correlation 0.4277 with marker block 227. In Tables 4 and 5, gene pairs mapped to the overly represented marker block 348 are given. We found that most of them have functions associated with mitochondrial-ribosome protein (*MRPS28*, *MPL22*, *RSM28*, *SWS2*), mitochondrial membrane or intermembrane space (*QCR6*, *SHE9*, *CYC1*), assembly of mitochondrial respiratory complexes (MBA1), intramitochondrial sorting(*YLR168C*) (see Table 5 for the gene by gene annotations). We further investigate genes within or near to the makerblock 348. We find *TIM54* very close to markerblock 348. Based on the Gene Ontology, *TIM54* is "Component of the mitochondrial Tim54p-Tim22p complex involved in insertion of polytopic proteins into the inner membrane" and localized in mitochondrial.

Table 1: The genome wide highest LA scores for 2D Linkage.

BOT				TOP			
Gene1	Gene2	Marker block	LA Score	Gene1	Gene2	Marker block	LA Score
YGR146C	YNL114C	227	-0.6842	YLR168C	CYC1	348	0.7097
ERR1	YGR018C	225	-0.6751	CYC1	MRPS28	348	0.6749
PDR16	ERR1	225	-0.6739	HMG1	YOR050C	186	0.6676
KRR1	YGR146C	227	-0.6629	SHE9	QCR6	348	0.6614
YGR146C	SAS10	227	-0.6625	YHR177W	YGR146C	225	0.6595
RPL36A	YPR063C	186	-0.6581	MUD1	MBA1	348	0.6591
YGR146C	RRB1	227	-0.6569	YOR146W	PAH1	301	0.6532
URB1	YGR146C	227	-0.6557	MRPL22	CYC1	348	0.6478
YEL077C	RRB1	407	-0.6511	QCR6	MRPS28	348	0.6475
GAS3	MAP1	301	-0.6495	SWS2	MUD1	348	0.6461
RPF1	YGR146C	227	-0.6488	YFR026C	YEL073C	576	0.6451
YGR146C	SER2	227	-0.6466	DUS1	YDR476C	510	0.6448
CPS1	YMR291W	510	-0.6445	CYC1	RSM28	348	0.6428
AKR2	RSM27	348	-0.6414	GCD2	ELG1	227	0.6421
RPL35A	YPR063C	186	-0.6414	FRE8	ERR3	225	0.6406
YGR146C	LTV1	227	-0.6413	TAF1	ADY2	227	0.64
SPT15	YPR063C	186	-0.6401	MRPS28	YGR235C	348	0.6398
NEW1	PFY1	123	-0.6401	YDR210W	ARP2	645	0.6393
SPO12	YML050W	172	-0.6394	YIL082W	RTS3	581	0.6385
HTS1	YGR146C	227	-0.639	GPM3	PAH1	301	0.6378

Table 2: Gene pairs mapped to the marker block 227 and the corresponding LA linkage scores.

Gene1	Gene2	Marker block	LA score
YGR146C	YNL114C	227	-0.6842
YGR146C	KRR1	227	-0.6629
YGR146C	SAS10	227	-0.6625
YGR146C	RRB1	227	-0.6569
YGR146C	URB1	227	-0.6557
YGR146C	RPF1	227	-0.6488
YGR146C	SER2	227	-0.6466
YGR146C	LTV1	227	-0.6413
YGR146C	HTS1	227	-0.639

Table 3: Annotations (from SGD) for genes linked to marker block 227 by 2D-trait mapping.

Gene	Annotation
YGR146C	Putative protein of unknown function; induced by iron homeostasis transcription factor Aft2p; multicopy suppressor of a temperature sensitive hsf1 mutant
KRR1	Essential nucleolar protein required for the **synthesis of 18S rRNA and for the assembly of 40S ribosomal subunit**
SAS10	**Component of the small (ribosomal) subunit (SSU) processosome required for pre-18S rRNa processing**; essential nucleolar protein that, when overproduced, disrupts silencing
RRB1	Essential nuclear protein **involved in early steps of ribosome biogenesis**; physically interacts with the ribosomal protein Rpl3p
URB1	Nucleolar protein required for the normal accumulation of 25S and 5.8S rRNAs, **associated with the 27SA2 pre-ribosomal particle**; **proposed to be involved in the biogenesis of the 60S ribosomal subunit**
RPF1	Nucleolar protein **involved in the assembly of the large ribosomal subunit**; constituent of 66S pre-ribosomal particles; contains a sigma(70)-like motif, which is thought to bind RNA
SER2	Phosphoserine phosphatase of the phosphoglycerate pathway, involved in serine and glycine biosynthesis, expression is regulated by the available nitrogen source
LTV1	Component of the GSE complex, which is required for proper sorting of amino acid permease Gap1p; **required for ribosomal small subunit export from nucleus**; required for growth at low temperature
HTS1	Cytoplasmic and mitochondrial histidine tRNA synthetase; encoded by a single nuclear gene that specifies two messages; efficient mitochondrial localization requires both a presequence and an amino-terminal sequence
YNL114C	Dubious open reading frame unlikely to encode a protein, based on available experimental and comparative sequence data; completely overlaps the verified ORF RPC19/YNL113W, an RNA polymerase subunit

Table 4: Gene pairs mapped to the marker block 348 and the corresponding LA linkage scores.

Gene1	Gene2	Marker block	LA score
CYC1	YLR168C	348	0.7097
CYC1	MRPS28	348	0.6749
CYC1	MRPL22	348	0.6478
CYC1	RSM28	348	0.6428
MRPS28	YGR235C	348	0.6398
MRPS28	QCR6	348	0.6475
SHE9	QCR6	348	0.6614
MUD1	SWS2	348	0.6461
MUD1	MBA1	348	0.6591

Table 5: Annotations (from SGD) for genes linked to marker block 348 by 2D-trait mapping.

Gene	Annotation
CYC1	Cytochrome c, isoform 1; electron carrier of the **mitochondrial** intermembrane space that transfers electrons from ubiquinone-cytochrome c oxidoreductase to cytochrome c oxidase during cellular respiration
YLR168C	Putative protein of unknown function that may be involved in **intramitochondrial sorting**; has similarity to Ups1p and to human PRELI; the green fluorescent protein (GFP)-tagged protein **localizes to mitochondria, r**equired for wild-type respiratory growth
MRPS28	**mitochondrial ribosomal small subunit component**
MRPL22	**Mitochondrial Ribosomal Protein, Large subunit**
RSM28	**Mitochondrial ribosomal protein** of the small subunit; genetic interactions suggest a possible role in promoting translation initiation
YGR235C	Putative protein of unknown function; the authentic, non-tagged protein is detected in highly purified **mitochondria** in high-throughput studies
QCR6	Subunit 6 of the ubiquinol cytochrome-c reductase complex, which is a component of the **mitochondrial inner membrane electron transport chain**; highly acidic protein; required for maturation of cytochrome c1
MUD1	U1 snRNP A protein, homolog of human U1-A; involved in nuclear mRNA splicing
MBA1	Protein involved in **assembly of mitochondrial respiratory complexes**; may act as a receptor for proteins destined for export from the mitochondrial matrix to the inner membrane
SWS2	**Putative mitochondrial ribosomal protein of the small subunit**, has similarity to E. coli S13 ribosomal protein; participates in controlling sporulation efficiency
SHE9	**Mitochondrial inner membrane protein** required for normal mitochondrial morphology, may be involved in fission of the inner membrane; forms a homo-oligomeric complex

Section C: eQTL Hotspots Obtained from Genome-wide Study

We further studied all the top 605 and bottom 1213 LA 2D-trait mapping results obtained using p-value cutoff 1e-5. The list of these 2D-trait mapping result can be found at http://kiefer.stat.ucla.edu/lap2/index-y-2g1m-genome-1e-5.htm. The marker block locations and their frequencies of occurrence are shown in Figure 4 in main text and in Table 6. The high frequency marker blocks are given in Table 7 along with a description of the enriched GO (gene ontology) term for the linked LA genes.

Table 6. Detected hotspot distribution by chromosome. The meanings of each column from left to right are chromosome number, total number of marker blocks within a chromosome, number of marker blocks detected by 2D mapping, number of hotspots with more than 20 occurrences of 2D-linked gene pairs and number of hotspots with more than 10 occurrences of 2D-lined gene pairs. Linkages with are listed separated according to the positive (TOP) and negative (BOT) LA scores. P-value gives the probability of observing a hotspot by pure chance.

chr	Total Marker Blocks	Mapped Marker Block Count (BOT)	Hotspots (≥20 linkages) (BOT) p=1e-15	Hotspots (≥10 linkages) (BOT) p=3e-6
I	21	3	0	0
II	48	3	0	0
III	14	1	0	0
IV	62	10	0	1
V	35	9	0	3
VI	25	9	1	2
VII	62	9	3	3
VIII	32	10	0	0
IX	32	11	2	2
X	46	13	1	1
XI	38	8	1	2
XII	64	10	0	0
XIII	52	16	1	2
XIV	32	2	0	0
XV	64	24	1	1
XVI	40	10	0	2
chr	Total Marker Blocks	Mapped Marker Block Count (TOP)	Hotspots (≥20 linkages) (TOP)p=8e-22	Hotspots (≥10 linkages) (TOP) p=4e-9
I	21	3	0	0
II	48	1	0	0
III	14	2	0	0
IV	62	5	0	1
V	35	9	0	0
VI	25	4	0	0
VII	62	10	2	3
VIII	32	3	0	0
IX	32	5	0	1
X	46	10	1	3
XI	38	4	1	1
XII	64	3	0	0
XIII	52	8	1	1
XIV	32	3	1	1
XV	64	13	0	1
XVI	40	3	0	0

Table 7. The mapped marker blocks whose occurrences (number of linked gene paired) exceed 20 were selected for GO enrichment analysis. Abbreviations: D= number of genes detected by LA scores; E=expected number of genes detected by chance. The column "candidate regulators" gives the genes that are either within the marker block or within the flanking regions (3kb) of the marker blocks. Those more relevant to the enriched GO terms are highlighted by bold typeface.

Top LA result

Num	Marker block	Freq	Enriched GO terms	Marker location	Candidate Regulators
1	225	28	phosphopyruvate hydratase activity D=8 E=0.04	chrVII : 256953-256953	**PCL10,** ITC1, RPL1B
2	227	98	nuclear lumen D=102 E=15.7	chrVII : 312740-318611	**SEH1, LSG1, RUF2**, **RPL28**, YGL102C, YGL101W
3	348	122	mitochondrial part D=148 E=15	chrX : 336317-345059	**TIM54**, MTR4, YJL049W, PEP8, YJL051W, TDH1
4	414	34	organellar ribosome D=11 E=0.84	chrXI : 643655-657365	**FLO10, NFT1**, YKR104W
5	510	53	isomerase activity D=26 E=0.88	chrXIII : 442910-445622	**YTA12**, VBA1, YMR087W, YMR086C-A
6	538	27	cell budding D=27 E=0.72	chrXIV : 191183-191243	ATG2

Bottom LA result

Num	Marker block	Freq	Enriched GO terms	Marker location	Candidate Regulators
1	186	79	cytosolic large ribosomal subunit D=56 E=2.2	chrVI : 5961-5964	COS4
2	225	75	phosphopyruvate hydratase activity D=42 E=0.12	chrVII : 256953-256953	**PCL10,** ITC1, RPL1B
3	227	142	nucleolus D=62 E=9.4	chrVII : 312740-318611	**SEH1, LSG1, RUF2**, **RPL28**, YGL102C, YGL101W
4	229	22	No enriched term	chrVII : 431864-435864	HOP2, AGA2, YGL034C, RPL24A, MIG1
5	301	42	eukaryotic 43S preinitiation complex D=35 E=1	chrIX : 9246-9276	YIL174W, YIL175W, YIL176C
6	307	30	cytosolic ribosome (sensu Eukaryota) D=44 E=1.5	chrIX: 46561-47053	**RPL40A**, TID3, AXL2, YIL151C, ECM37, CCT2, MCM10, TPM2, REV7, PAN6, MLP2, SSL2, SLN1, YIL141W
7	348	66	organellar ribosome D=24 E=1.6	chrX : 336317-345059	**TIM54**, MTR4, YJL049W, PEP8, YJL051W, TDH1
8	407	296	ribonucleoprotein complex biogenesis and assembly D=268 E=33	chrXI : 522777-522777	**UIP5,** PET10, YKR047W, YKR045C, YKR043C
9	510	58	isomerase activity D=9 E=1	chrXIII : 442910-445622	**YTA12**, VBA1, YMR087W, YMR086C-A
10	575	27	cytosolic ribosome (sensu Eukaryota) D=9 E=1.4	chrXV : 180210-180961	**REX4**, AVO1, YOL079W

Table 8. Annotations for some candidate regulators in Table 6

Gene	Annotation
FLO10	Lectin-like protein with similarity to Flo1p, thought to be involved in flocculation
LSG1	Putative GTPase involved in 60S ribosomal subunit biogenesis; required for the release of Nmd3p from 60S subunits in the cytoplasm
NFT1	Putative transporter of the multidrug resistance-associated protein (MRP) subfamily
PCL10	Biological Processes are regulation of glycogen biosynthetic process, regulation of glycogen catabolic process
REX4	Putative RNA exonuclease possibly involved in pre-rRNA processing and ribosome assembly
RPL28	Ribosomal protein of the large (60S) ribosomal subunit
RPL40A	Ribosomal Protein of the Large subunit
RUF2	H/ACA box small nucleolar RNA (snoRNA); guides pseudouridylation of large subunit (LSU) rRNA at positions U1110, U2349, and U2351
SEH1	Nuclear pore protein that is part of the evolutionarily conserved Nup84p complex (Nup84p, Nup85p, Nup120p, Nup145p, and Seh1p); homologous to Sec13p
TIM54	Component of the mitochondrial Tim54p-Tim22p complex involved in insertion of polytopic proteins into the inner membrane
UIP5	interacts with Ulp1p, a Ubl (ubiquitin-like protein)-specific protease for Smt3p protein conjugates
YTA12	Component, with Afg3p, of the mitochondrial inner membrane m-AAA protease that mediates degradation of misfolded or unassembled proteins and is also required for correct assembly of mitochondrial enzyme complexes

Section D: 1D-trait mapping results for gene pairs identified by genome-wide 2D-trait mapping

We studied the 1D linkages of the 1818 LA 2D-trait mapping results obtained using p-value cutoff 1e-5. We found that the vast majority of these 2D-trait mapping results cannot be identified by 1D-trait mapping. Specifically, each 2D-trait mapping result is a triplet (X, Y, Z) where X and Y are two gene expression traits and Z is a marker block. We used absolute correlation to measure the relation between one gene expression trait and one marker, and used $\rho = \max\{|\mathrm{corr}(X,Z)|, |\mathrm{corr}(Y,Z)|\}$ to measure whether this triplet can be at least partially identified by 1D-trait mapping. Figure 4 (b) shows the distribution of ρ for the 1818 LA 2D-trait mapping results. The correlations are generally quite low, with median 0.13 and 95 percentile 0.27. In contrast, significant 1D linkages correspond to much higher absolute correlations with median 0.68 and the 5 percentile is 0.59 (Figure 4 (c)). We also show the distribution of $\rho_1 = \min\{|\mathrm{corr}(X,Z)|, |\mathrm{corr}(Y,Z)|\}$ in Figure 4 (a). Comparing with ρ, ρ_1 measures whether the 2D result can be completely identified by 1D mapping. As expected, ρ_1 is quite small, with median 0.06 and 95 percentile 0.16.

Figure 4. (a) The distribution of $\rho_1 = \min\{|\mathrm{corr}(X,Z)|,|\mathrm{corr}(Y,Z)|\}$ for 1818 triplets (X,Y,Z) obtained by LA 2D-trait mapping, where X and Y are two gene expression profiles and Z is the corresponding marker block.
(b) The distribution $\rho = \max\{|\mathrm{corr}(X,Z)|,|\mathrm{corr}(Y,Z)|\}$ for 1818 triplets (X,Y,Z) obtained by LA 2D-trait mapping.
(c) The distribution of the absolute correlations corresponding to 231 significant 1D linkages. These 231 significant linkages are taken from eight linkage hotspots reported by Brem et al. (2002).

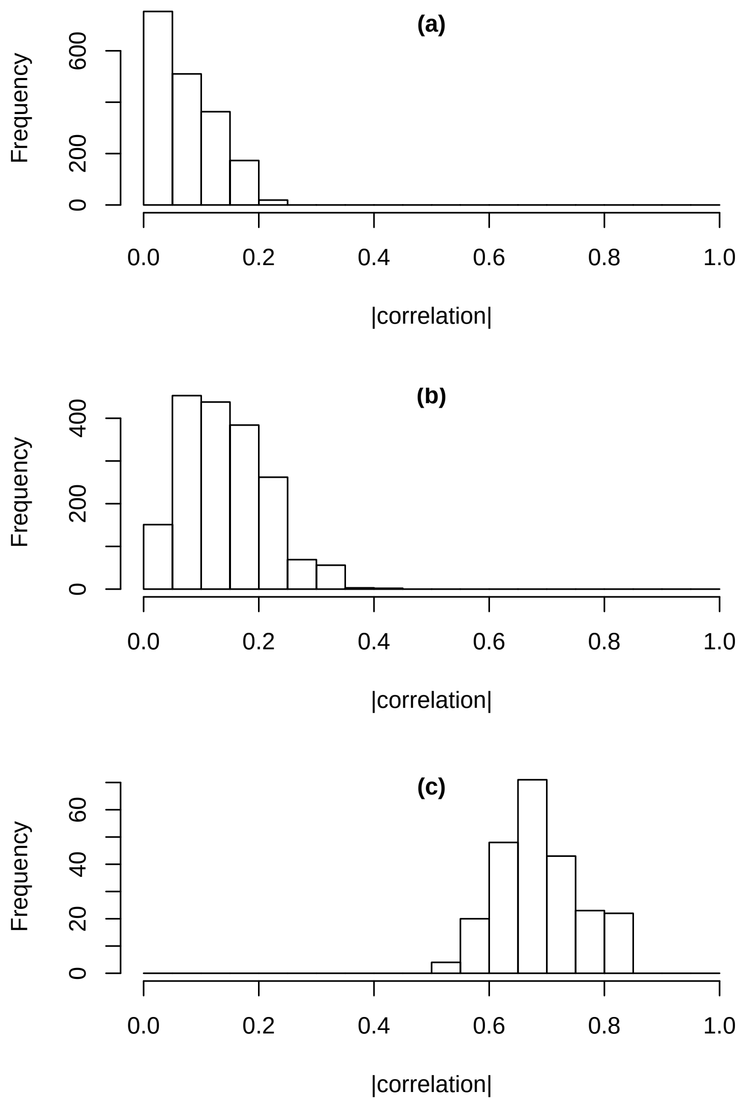

Supplement: Additional file 2 — Supplementary Materials, Part II. Supplementary genome-wide results of 2D-trait mapping. [file 1471-2164-9-242-S2.pdf]
